# MRI in Neurosciences

**DOI:** 10.1155/2012/579192

**Published:** 2012-05-20

**Authors:** Alayar Kangarlu, Ramin V. Parsey, Eric C. Bourekas

**Affiliations:** ^1^Department of Psychiatry, Columbia University, NewYork, NY 10032, USA; ^2^Department of Radiology, The Ohio State University, Columbus, OH 43210, USA

Magnetic Resonance (MR) has empowered neuroscience with a tool to investigate the inner structure and workings of the central nervous system (CNS). Unlike many imaging techniques, MR offers multiple modalities in one package, enabling scientists access to a wide range of unknowns from microstructure to physiology of the brain. In this issue, developments in structural and functional MR imaging, spectroscopy, image processing, and applications are presented that could expand MRI's ability in unraveling some of the mysteries of the brain. These works demonstrate that in spite of its rapid growth MRI still has much room for further development and penetration in neuroscience research. 

Dong et al. address two of the major challenges in spectroscopy, that are, whole brain coverage and sensitivity. They present a technique for taking advantage of multichannel technology, a powerful signal detection scheme, to increase sensitivity of MR spectroscopy of the brain. Furthermore, Dong et al. offer a multiplanar approach for whole brain coverage, while taking advantage of multichannel radio frequency coils to increase signal-to-noise ratio in order to make whole brain spectroscopy a viable tool for metabolite detection. Dr Zhang, in the second paper, takes up multiple sclerosis (MS), which soon after the invention of MRI, was perceived to benefit the most among all CNS diseases from the high soft-tissue contrast of this technique. In spite of its ability to acquire exquisite images of demyelinating plaques, MRI has not fulfilled its undeclared promise of achieving imaging-based diagnosis in MS. In his review, Dr Zhang draws attention to the higher order sensitivities of MR images, that are, inner voxel signal variation or texture, to highlight the heterogeneous nature of MS. He offers an image analysis technique to pick up variations in plaque intensity patterns that at the same time can quantify pathological changes. This technique could become a sensitive tool in search for the pathogenesis and in monitoring progression of this disease. Dr N. Robitaille and colleagues have taken up the issue of standardization of images acquired in multicenter studies. To take MRI to the next stage of validation of findings for clinical applications, isolated findings have to be reproduced by geographically-separated teams using different hardware. Such multicenter studies help strengthen the merits of findings and ultimately expand the scope of MRI in its neurological applications. These investigators offer an intensity standardization technique to correct scanner-dependent intensity variations, something that every participant of multicenter studies has suffered from and for which everyone will welcome a solution. Another paper of this issue offers a novel automatic technique, which is both simple and robust and able to use tissue-spatial intensity information to forge a more sensitive measure of brain-tissue assessment. The following paper is also from the Robitaille group and they bring into focus medical image segmentation. Finding the best way to combine different measures of the image labeling they survey the primary labeling techniques and forge a hybrid of them called SVS which is a “label fusion strategy” that they show being superior to any of the methods examined. They have used segmentations in challenging areas of the brain, that are, hippocampus and amygdala, to prove their point and validate their finding. A. Borogovac and I. Asllani chose an emerging MRI technology to highlight in this issue. Arterial spin labeling (ASL) is fast gaining acceptance in its ability to detect cerebral blood flow (CBF) and reliably establish correlation with brain function. ASL has proven to be able to offer meaningful fMRI-based surrogates of brain function, manifested in terms of hemodynamics. This could serve research on healthy and diseased brain, in both neuroscience and neurology. While on the issue of fMRI, we will read our next contribution from T. Christensen et al. staging the power of this technique in assessing the nature of attention and its role in encoding information into memory. This group shows the power of fMRI in demonstrating how an imaging technique can quantitatively probe attention and what it takes to offer an account of contributions from recollection memory and incidental-perceptual memory. fMRI revealed that BOLD was modulated differentially by unintentionally encoded words compared to novel items. Such use of MRI in providing evidence in support of models of memory consolidation and retrieval is a confirmation of the power of this technique in exploring neuronal processes. Finally, Y. Liu et al. discuss their technique for automation of landmark selection in image registration. Automation of registration of MRI with histology images or EPI with high-resolution anatomical images will allow the images in one modality to benefit from information contained in another. Accurate registration achieved by automatically generated landmarks will help interpretation of images from high-field rodent images, as well as high-distortion images such as EPI acquired from human scanners.

This special issue hoped to highlight “MRI in Neuroscience.” Representative research reports covering the latest advancements in magnetic resonance acquisition or image processing, applications in neuroscience research, and clinical neurological applications are presented in this issue. These and similar works are rapidly expanding the use of MRI in neuroscience and through this encouraging more investigators to use this powerful tool in their research. 

*Alayar Kangarlu* *Alayar Kangarlu* *Ramin V. Parsey* *Ramin V. Parsey* *Eric C. Bourekas* *Eric C. Bourekas*

